# Green synthesis of silver nanoparticles mediated *Diospyros kaki* L. (Persimmon): determination of chemical composition and evaluation of their antimicrobials and anticancer activities

**DOI:** 10.3389/fchem.2023.1187808

**Published:** 2023-05-30

**Authors:** Cumali Keskin, Ali Ölçekçi, Ayşe Baran, Mehmet Fırat Baran, Aziz Eftekhari, Sabina Omarova, Rovshan Khalilov, Elvin Aliyev, Albert Sufianov, Aferin Beilerli, Ilgiz Gareev

**Affiliations:** ^1^ Department of Medical Services and Techniques, Vocational School of Health Services, Mardin Artuklu University, Mardin, Türkiye; ^2^ Department of Food Technology, Vocational School of Technical Sciences, Batman University, Batman, Türkiye; ^3^ Department of Biochemistry, Faculty of Science, Ege University, Izmir, Türkiye; ^4^ Department of Biophysics and Biochemistry, Baku State University, Baku, Azerbaijan; ^5^ Department of Biology and Ecology, Faculty of Natural sciences, Lankaran State University, Lankaran, Azerbaijan; ^6^ Educational and Scientific Institute of Neurosurgery, Peoples’ Friendship University of Russia (RUDN University), Moscow, Russia; ^7^ Department of Neurosurgery, Sechenov First Moscow State Medical University (Sechenov University), Moscow, Russia; ^8^ Department of Obstetrics and Gynecology, Tyumen State Medical University, Tyumen, Russia; ^9^ Central Research Laboratory, Bashkir State Medical University, Ufa, Bashkortostan, Russia

**Keywords:** *Diospyros kaki*, gold nanoparticles, antimicrobial, anticancer, plant based nanoparticles

## Abstract

The eco-friendly synthesis of metallic nanoparticles (MNPs) using biological materials is an encouraging and innovativeness approach to nanotechnology. Among other synthesizing methods, biological methods are chosen because of their high efficiency and purity in many aspects. In this work, using the aqueous extract obtained from the green leaves of the *D. kaki* L. (DK); silver nanoparticles were synthesized in a short time and simply with an eco-friendly approach. The properties of the synthesized silver nanoparticles (AgNPs) were characterized using various techniques and measurements. In the characterization data of AgNPs, Maximum absorbance at 453.34 nm wavelengths, the average size distribution of 27.12 nm, the surface charge of −22.4 mV, and spherical appearance were observed. LC-ESI-MS/MS analysis was used to assess the compound composition of D. kaki leaf extract. The chemical profiling of the crude extract of D. kaki leaves revealed the presence of a variety of phytochemicals, predominantly phenolics, resulting in the identification of five major high-feature compounds: two major phenolic acids (Chlorogenic acid and Cynarin), and tree flavonol glucosides (hyperoside, quercetin-3-glucoside, and quercetin-3- D-xyloside). The components with the highest concentrations were cynarin, chlorogenic acid, quercetin-3- D-xyloside, hyperoside, and quercetin-3-glucoside, respectively. Antimicrobial results were determined by a MIC assay. The biosynthesized AgNPs exhibited strong antibacterial activity against the human and food pathogen Gram (+ and −) bacteria and good antifungal activity against pathogenic yeast. It was determined that 0.03–0.050 μg/mL concentrations ranges of DK-AgNPs were growth suppressive concentrations on all pathogen microorganisms. The MTT technique was used to study the cytotoxic effects of produced AgNPs on cancer cell lines (Glioblastoma (U118), Human Colorectal Adenocarcinoma (Caco-2), Human Ovarian Sarcoma (Skov-3) cancer cell lines, and Human Dermal Fibroblast (HDF) healthy cell line). It has been observed that they have a suppressive effect on the proliferation of cancerous cell lines. After 48 h of treatment with Ag-NPs, the DK-AgNPs were found to be extremely cytotoxic to the CaCo-2 cell line, inhibiting cell viability by up to 59.49% at a concentration of 50 g mL^−1^. It was found that the viability was inversely related to the DK-AgNP concentration. The biosynthesized AgNPs had dose-dependent anticancer efficacy. Because of the high concentration of bioactive chemicals in *Diospyros kaki*, it may be employed as a biological resource in medicinal applications. DK-AgNPs were shown to be an effective antibacterial agent as well as a prospective anticancer agent. The results provide a potential approach for the biogenic production of DK-AgNPs utilizing *D. kaki* aqueous leaf extract.

## Introduction

**FIGURE 1:**
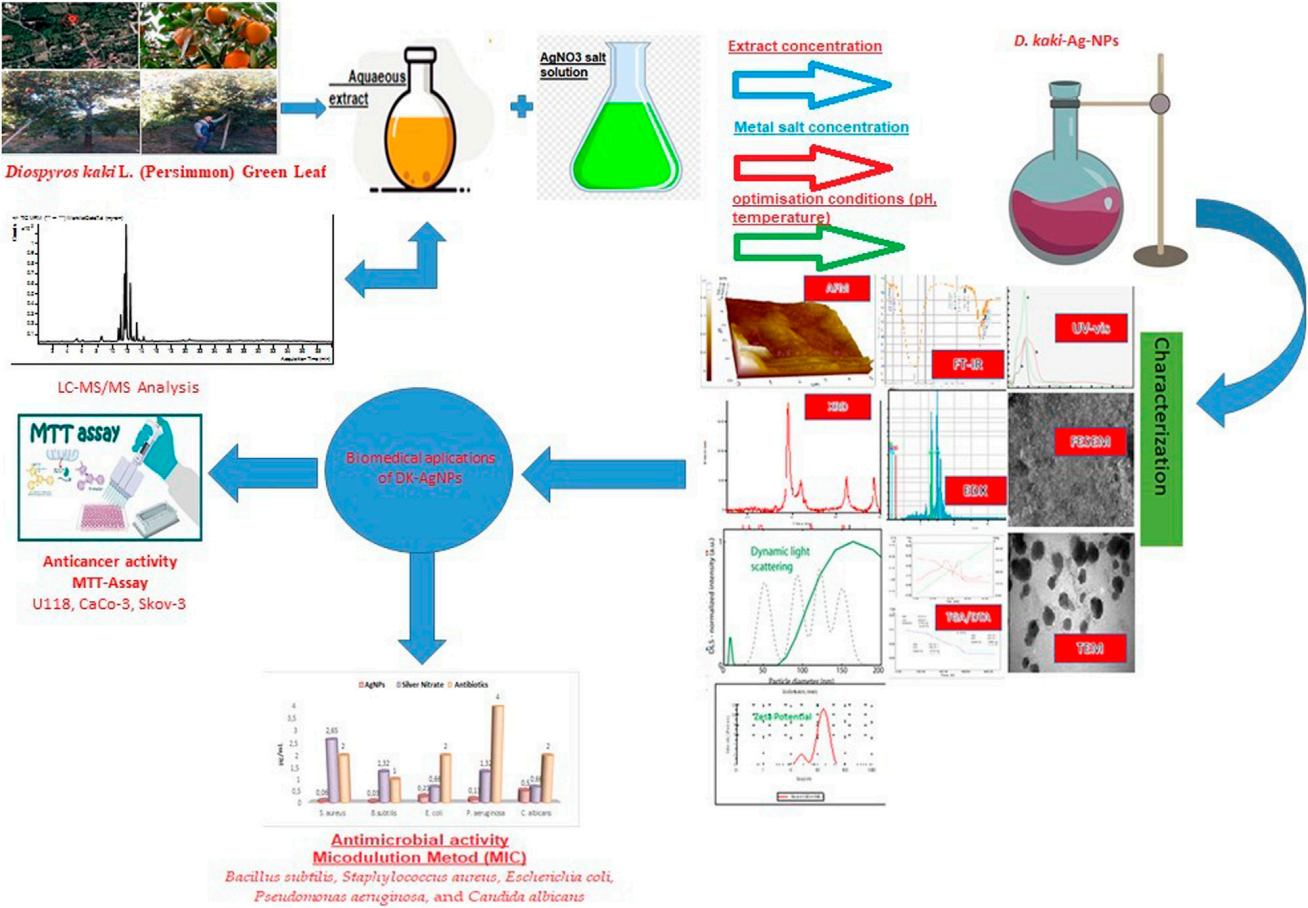
Schematic representation of synthesis, characterization, and biomedical applications of silver nanoparticles synthesized using *D. kaki* leaf aqueous extract.

Green nanotechnology, which is included in nanotechnology, has recently attracted great attention with the elements it reveals at the molecular level. These products continue to be a developing field of study with their use in the treatment of various diseases in medicine and many areas (Ramazanli and Ahmadov, 2022). Nanoparticles (NPs) have superior properties such as physical and chemical. In addition to the large surface areas of NPs, their resistance to high-temperature changes are among their superior features (Syafiuddin et al., 2017). In particular, nanometals such as gold (Au), zinc (Zn), silver (Ag), palladium (Pd), and titanium (Ti) is used in areas such as drug delivery systems, biological labeling, optical devices, and some processes such as the synthesis and stability of metal nanoparticles (NPs) attract great attention in this field (Emmanuel et al., 2015). In addition, NPs are valuable materials with uses in biomedical applications, cosmetics, and food industries, bioremediation studies, etc. (Khalilov, 2023; Arroyo et al., 2020). Different methods are used to obtain metallic NPs. Among these methods, synthesis by biological methods has some advantages. Among these are advantages such as being eco-friendly, nominal energy, and cost, as it does not contain toxic chemicals in the synthesis stages (Singh et al., 2018). Long before antibiotics were brought into contemporary medicine, silver was used to cure infected wounds. However, because of its high toxicity and the availability of antibiotics, the use of silver is restricted. Recently, silver nanoparticles (AgNPs) have piqued the interest of scientists due to their potential application as antibacterial agents (Gunashova, 2022). Because of their broad bactericidal and fungicidal spectrum, they have a wide range of uses in medicine, including medicines, cosmetics, and medical devices. The most pressing public health issues for physicians have been antibiotic resistance and anti-cancer agents that may be administered at low dosages to inhibit malignant cell proliferation. As a result, research into employing nanotechnology to improve medication delivery has begun (Hawar et al., 2022). There are many studies using plant sources (leaf, flower, root, fruit, or whole plant) to obtain silver nanoparticles (AgNPs) with biological approaches (Namburi et al., 2021). AgNPs obtained in synthesis studies with plant sources do not require special conditions and are synthesized with an eco-friendly approach, as well as the fact that the synthesis process is easy, and cheap and the amount of product is higher, among the factors that increase the interest in biological approaches (Rather et al., 2022). Plants are the finest alternatives for finding novel anti-cancer chemicals that have fewer side effects and are more affordable. Infectious diseases, cancer, pyrexia, algesia, and inflammation can all be treated using a variety of therapeutic substances that can be found in medicinal plants (Majid et al., 2022a; Majid et al., 2022b). Phenolic compounds, alcohols, flavonoids, and phytochemicals containing carboxyl groups in the extract obtained from plant sources are compounds that form silver nanoparticles by reducing the plus-valent silver in the aqueous structure and also have an effect on stability (Srikar et al., 2016). AgNPs are used in medical applications as antimicrobial (Rather et al., 2022) and anticancer (Salman et al., 2022) agents, in cosmetics, bioremediation studies (Rani et al., 2020), in the food industry (Velmurugan et al., 2014), and in many other areas. *Diospyros kaki* L. (Persimmon of Paradise) plant, which has about 400 members of the Ebenaceae family, is distributed throughout Asia, Africa, and Central and South America. It is an economically valuable plant. The leaves of the plant have medicinal uses with their positive effect in the treatment of paralysis, skin burns, stopping bleeding, and frostbite. It is chemical composition is rich in variable bioactive components such as phenolic compounds, flavonoid oligomers, tannins, ascorbic acid, and caffeine (Çifçi et al., 2021). In this work, AgNPs were produced utilizing the aqueous extract of *D. kaki* leaf. AgNPs that were produced in the plant-based green method were assessed for two biological activities. Two Gram-positive, two Gram-negative, and one fungus were used in the current study to evaluate the antibacterial properties of biogenic AgNPs. Four cell lines were employed in the study to determine cytotoxicity. By using LCMS-MS to analyze the chemical composition of the leaf extract, it was possible to identify probable biologically active groups. The novelty of the present study is that it successfully highlighted the antimicrobial and cytotoxic potential of AgNPs synthesized for the first time using *D. kaki* leaf aqueous extract using different cell lines, with abundant evidence (Figure 1).

## Materials and methods

### Extraction

The leaves of *D. kaki* (DK) were collected close to shedding in late November. The leaves were washed three times with deionized water to eliminate any pollutants and dirt. The plants were then diced and dried in the open air until they reached a consistent weight. Following that, dried leaves were pulverized into a fine powder in a grinder. 150 grams of the resulting powder were combined with 500 mL of deionized water before being heated at 85°C and swirled at 240 rpm with a magnetic stirrer for around 60 min. The heated solutions were eventually filtered, and the filtrate extracts were kept in a glass beaker at 4.0°C for further use.

### Synthesis process of DK-AgNPs

5 mM (millimolar) metal solution was prepared by using silver nitrate (99.8% purity), ACS reagent, ≥99.0% (Sigma Aldrich) for the synthesis of DK-AgNPs. DK the leaf extract and AgNO_3_ solution (1:4) were mixed at 30°C for synthesis. The colour change was observed in 15 min. Depending on the colour change, samples were taken from the reaction medium.

### Characterization process of DK-AgNPs

UV-Vis spectroscopy is an essential method for assessing the synthesis and stability of metal nanoparticles in aqueous solutions. The maximum absorbance value does not change when samples taken at various periods are analyzed, which is the stability criteria (Jabir et al., 2021). Under optimum circumstances, a 50 mL AgNP colloid solution was prepared with 5 mM silver nitrate and centrifuged at 15,000 revolutions per minute for 10 min for Fourier transform infrared (FTIR) spectroscopy analysis. After that, the pellets were resuspended and lyophilized for 24 h. FTIR analysis utilizing (Perkin Elmer Spectrum one) may be used to determine the various functional groups in the generated Ag nanoparticles. It provides information on the structure of a molecule, which is frequently obtained through absorption spectra (Keskin et al., 2022). The crystal patterns and crystal sizes of the particles formed as a result of the synthesis were analyzed using an XRD. Using the results obtained with XRD at 2θ in the range of 20–80, the crystal nano dimensions of AgNPs formed after synthesis was calculated by the Debye-Scherrer formula (Keskin et al., 2022). Morphological appearances of synthesized AgNPs were determined using TEM micrograph images with EVO 40 LEQ SEM, and Jeol Jem 1010 FESEM instruments. Topographic features and phase contrast structure, morphological appearance, and size of DK-AgNPs were determined using the AFM (Park System XE-100 AFM) device. In addition, the data obtained from TEM and SEM images were also used for morphological structure evaluation. Surface charges and size distributions of synthesized DK-AgNPs were measured by Zeta potential distribution (Malvern) device.

### Determination of chemical composition of plant extract

To determine the phytochemicals that may be responsible for bioreduction, the phytochemical profile of the extracted content was defined using liquid chromatography-mass spectrometry (LC-MS/MS) and FTIR devices. The results were also used to determine the chemical components of the extract.

### Antimicrobial activities of biogenic AgNPs

The suppressive effects of AgNPs synthesized from the extract of DK leaves on the growth of pathogenic microorganism strains were determined according to the minimum inhibition concentration (MIC) value using the microdilution method (Keskin et al., 2022). Gram-positive *B. subtilis* ATCC 11774 (*Bacillus subtilis*) and *Staphylococcus aureus* ATCC 29213 (*S. aureus*) and Gram-negative *P. aeruginosa* ATCC27833 (*Pseudomonas aeruginosa*) and *E. coli* (*Escherichia coli*) ATCC25922 bacterial strains and *Candida albicans* yeast (*C. albicans*) were used to determine antipathogenic activities. Standard antibiotics (vancomycin, colistin, and fluconazole) used to compare the effects of biogenic nanoparticles were commercially purchased from Sigma Aldrich. Solutions containing AgNPs at 1 μg/mL concentration were prepared and transferred to microplates in appropriate amounts, and AgNPs were dispersed in the medium with a series of micro dilutions. Microorganisms grown on the medium plates were taken and solutions containing microorganisms prepared by the McFarland 0.5 (Emmanuel et al., 2015; Maillard et al., 2018; Nishanthi et al., 2019) turbidity criteria were transferred. Some microplate wells were defined for control steps such as sterilization. After all these procedures, the prepared microplates were incubated at 37°C. It was incubated overnight (24 h) for antimicrobial interaction. The next day, the microplate wells were checked for the proliferation of microorganisms, and the MIC was determined.

### Cytotoxic effects of biogenix AgNPs on healthy and cancerous cell lines

Cytotoxic effects of synthesized AgNPs and their suppressive effects on cancer cells and healthy cells were investigated using the MTT method (Khatamifar et al., 2022) at Dicle University Scientific Research Center, Diyarbakır, Turkey. In the experimental study, the effects of AgNPs on Glioblastoma (U118), Human Colorectal Adenocarcinoma (Caco-2), Human Ovarian sarcoma (Skov-3) cancer cell lines, and Human Dermal Fibroblast (HDF) healthy cell lines were studied. In 75 t-flasks of Dulbecco’s Modified Eagle (DMEM) medium, cell lines HDF, Caco-2, and U118 were cultured. DMEM medium contains pentrep, 2 mM L-Glutamine, and 10% FBS. The Skov-3 cell line was cultured in 75 t-flasks in RPMI media with 100 U/mL penstrep and 10% FBS. The cultured flasks were stored in a 37°C oven with 95% air, 5% CO_2_, and humidity. The cell lines were then measured using a hemocytometer and resuspended at varying concentrations depending on whether or not they were at 80% confluence. Cell lines were then incubated overnight in 96-well microplates (24 h). After processing, varied doses of AgNPs were introduced to the wells where the cell lines were cultivated, and the oven was set to 37°C for 48 h. After the interval, MTT solution was added to the wells and the microplates were incubated for 3 h. After applying DMSO and waiting 15 min, absorbance measurements of the cells at 540 nm wavelength were collected using a Multi Scan Go, Thermo equipment. The equation below was used to determine AgNPs generated with cell line absorbance values, which inhibit their viability in cell lines (Remya et al., 2015).

$$\%\;Viability=\frac{the\;absorbance\;of\;cells\;after\;exposure\;to\;AgNPs}{the\;absorbance\;of\;the\;control\;cells}X100$$ (1)

### Statistical analysis

The obtained data were statically analyzed using an unpaired *t*-test with GraphPad Prism. The values were presented as the mean ± SD (*n* = 3) (Ali et al., 2018).

## Results and discussion

### UV-vis spectrum data of DK-AgNPs

**FIGURE 2:**
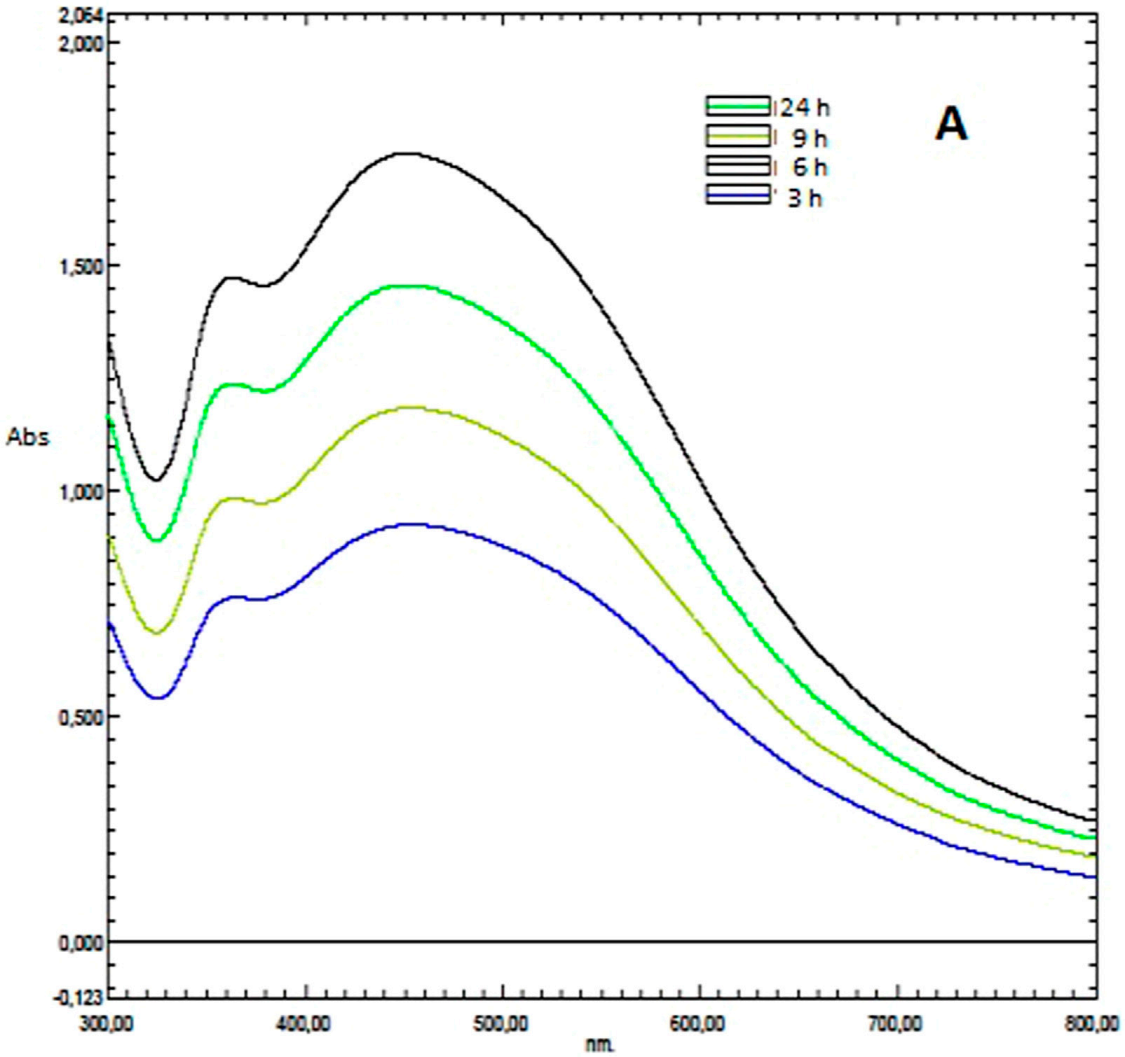
**(A)** Maximum wavelength absorbance bands showing the presence of AgNPs synthesized by leaf extract.

UV-vis spectrum (Figure 2) illustrates the influence of silver nitrate concentration during the creation of silver nanoparticles using *D. kaki* leaf extract. Brown-colored silver nanoparticles made from 1 mM silver nitrate were found to have a distinctive surface plasmon absorption band at 453 nm. While other concentrations displayed a spacious peak at 453 nm, the 1 mM concentration exhibited a narrow band with enhanced absorbance (Figure 2). When the number of silver ions was raised from 1 to 5 mM, the absorption rate increased. Due to the greater availability of functional groups in the leaf extract at 1 mM concentration, the formation of nanoparticles and size reduction got underway right away. Due to competition between the functional groups in the 10 mL leaf extract and the silver ions, as the substrate concentration was increased, the enormous size and aggregation of nanoparticles occurred. Therefore, a considerable impact of concentration on the production of silver nanoparticles was demonstrated by the optimization investigation. According to the results of this experiment, a concentration of 1 mM silver nitrate was ideal for the creation of nanoparticles. Similar to intensity, an increased concentration of nanoparticles is indicated by increasing intensity. A higher silver nitrate concentration may indicate the creation of bigger nanoparticles.

### XRD data of DK-AgNPs

**FIGURE 3:**
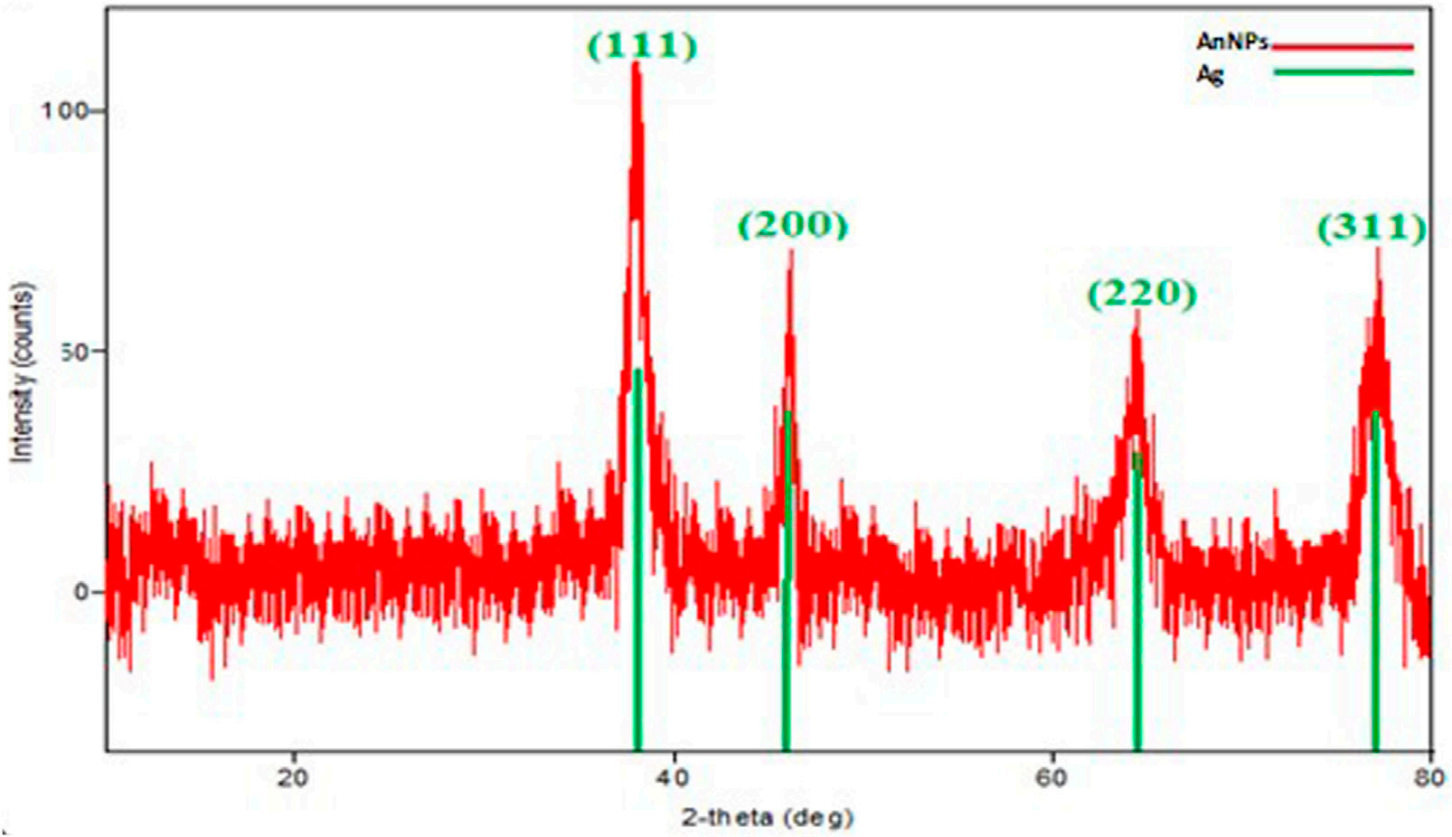
Maximum wavelength absorbance bands showing the presence of AgNPs synthesized by leaf extract.

The characteristic peaks in the XRD spectra validated and demonstrated the XRD pattern of AgNPs formed from the leaf extract (Figure 3). The spectra taken at 111°, 200°, 220°, and 311° in the analysis data were performed at 2θ to elucidate the crystal structures of the particles synthesized with DK leaf extract and to determine the crystal nano sizes showed that the particles had a cubic (face-centered cubic; JCPDS File No. 04–0783) pattern. Four Bragg’s reflexion patterns at 2θ, that is, 111.00°, 200.00°, 220.00°, 311.00°, and in the whole spectrum of values ranging from 37 to 78, were interpreted from XRD. Using the values of these spectra, the crystal nano dimensions of AgNPs were calculated as 48.90 nm using the Debye-Scherrer equation (Khan et al., 2018). Using this equation, it was stated that the crystal nano dimensions of AgNPs were calculated as 27.30 nm (Some et al., 2019) and 39.37 nm (Baran, 2019) in Eco-Friendly synthesis studies.

### Morphologies of biogenic AgNPs

**FIGURE 4:**
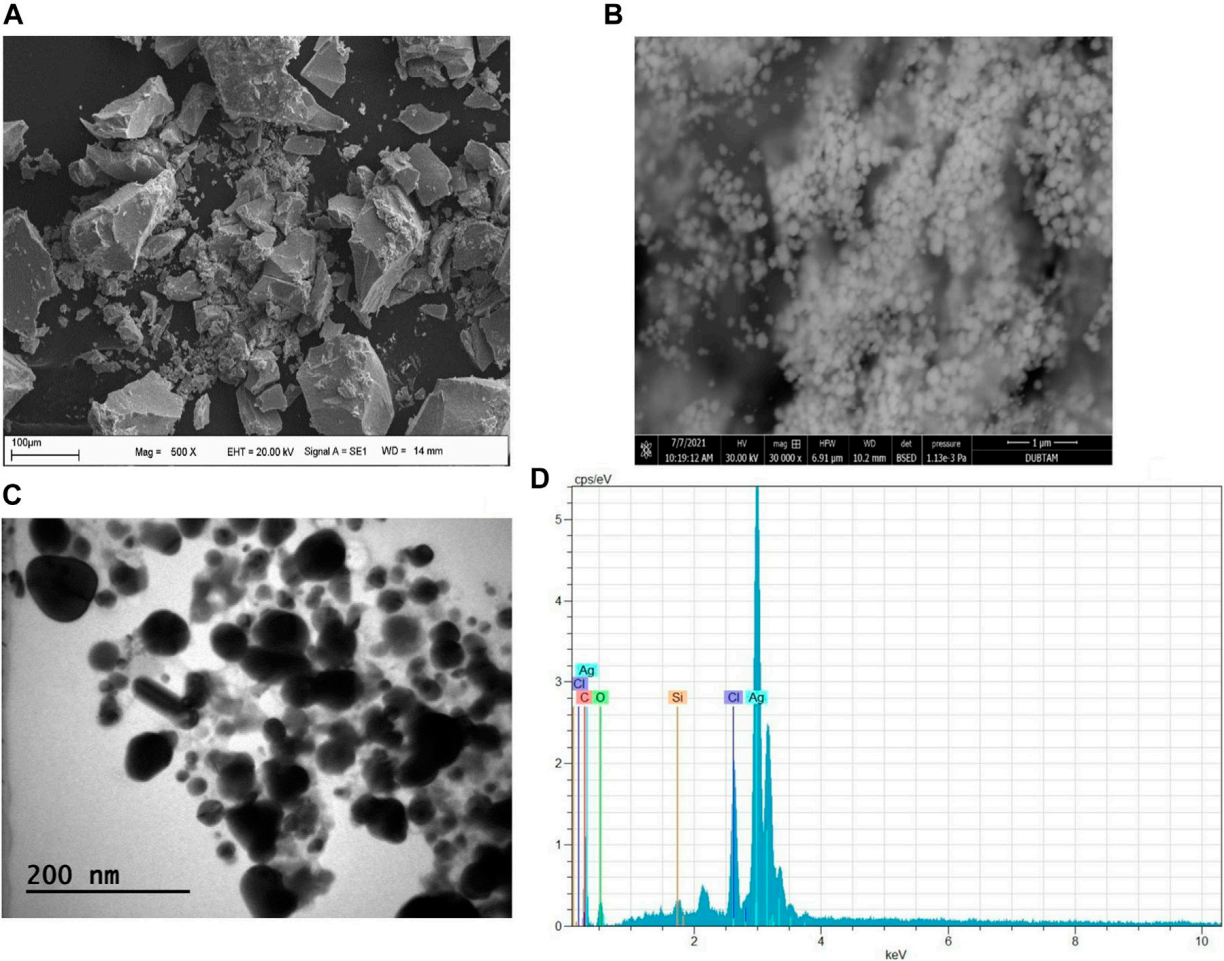
AgNPs synthesized by DK extract; **(A)** SEM, **(B)** FESEM, **(C)** TEM micrographs, and **(D)** EDX Profile.

SEM, FESEM, and TEM micrograph analysis results used to define the morphological structures of the synthesized AgNPs were spherical in appearance and 8.13–38.5 nm in size (Figure 4). In a study, it was reported that AgNPs obtained by mixing Pine, Ginkgo, Persimmon, Magnolia, and Platanus plant extracts were spherical in SEM images (Song and Kim, 2009). In another biosynthesis study, micrographs of the spherical morphological appearance of AgNPs were presented (Hasanzadeh et al., 2021). The same findings were expressed in the FESEM results of AgNPs synthesized in the environmentally friendly synthesis study of *Sterculia foetida* leaf extract (Premkumar et al., 2018). Previous research, FESEM for Phyto-synthesised silver nanoparticles, strongly validated this evaluation of AgNPs analyzed using the FESEM approach (Khashan et al., 2020). The TEM is used to evaluate the morphological properties of the produced NPs (TEM) (Al-Musawi et al., 2020). From TEM images obtained in a study in which AgNPs were synthesized and characterized (Kumar et al., 2017), it has been reported that nanoparticles have a spherical appearance and dimensions of 12–50 nm. In the EDX analysis to determine the elemental content of the synthesized particles, strong peaks of silver in Figure 4 showed the presence of AgNPs. Analysis using an Energy Dispersive X-ray (EDX) spectrometer verified the occurrence of silver’s elemental signal and its uniform pattern of dispersion of nanoparticles. The horizontal axis showed energy in KeV, whereas the vertical axis showed the quantity of X-ray counts. This confirmed that silver particles were appropriately identified and is present in the solution. Identification lines for the main silver emission energies were displayed, and these coincided with peaks in the spectra. In addition to these strong peaks, the presence of weak peaks such as carbon and oxygen was also due to the phytochemicals in the particles (Khatamifar et al., 2022).

### AFM micrograph of biogenic AgNPs

**FIGURE 5:**
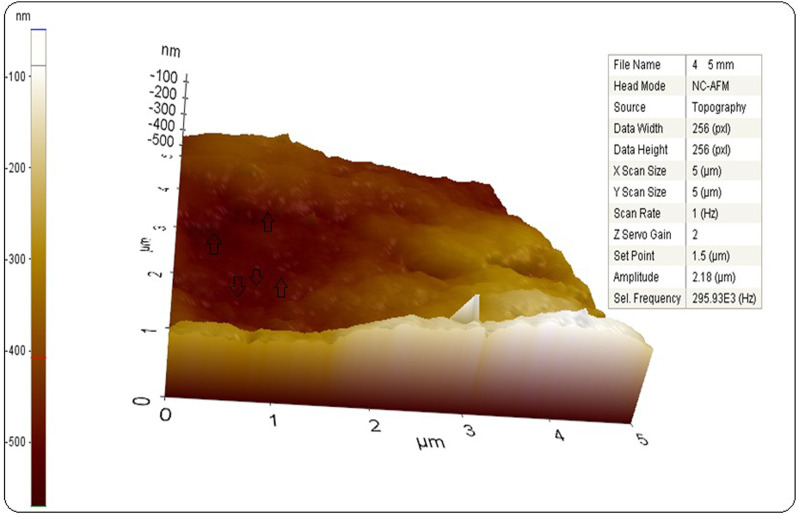
AFM graph of synthesized AgNPs.

The topographic distributions and size distributions of AgNPs obtained as a result of the Eco-Friendly method were determined by the results of AFM analysis. As seen in Figure 5, it was seen that The Atomic Force Microscopy (AFM) image depicts monodispersed silver nanoparticles that were created, and it agrees well with the SEM and TEM images. In the AFM results of similar studies, the data showing that AgNPs have a spherical appearance and are below 100 nm were examined (Khashan et al., 2020).

### Zeta potential distribution of DK-AgNPs

**FIGURE 6:**
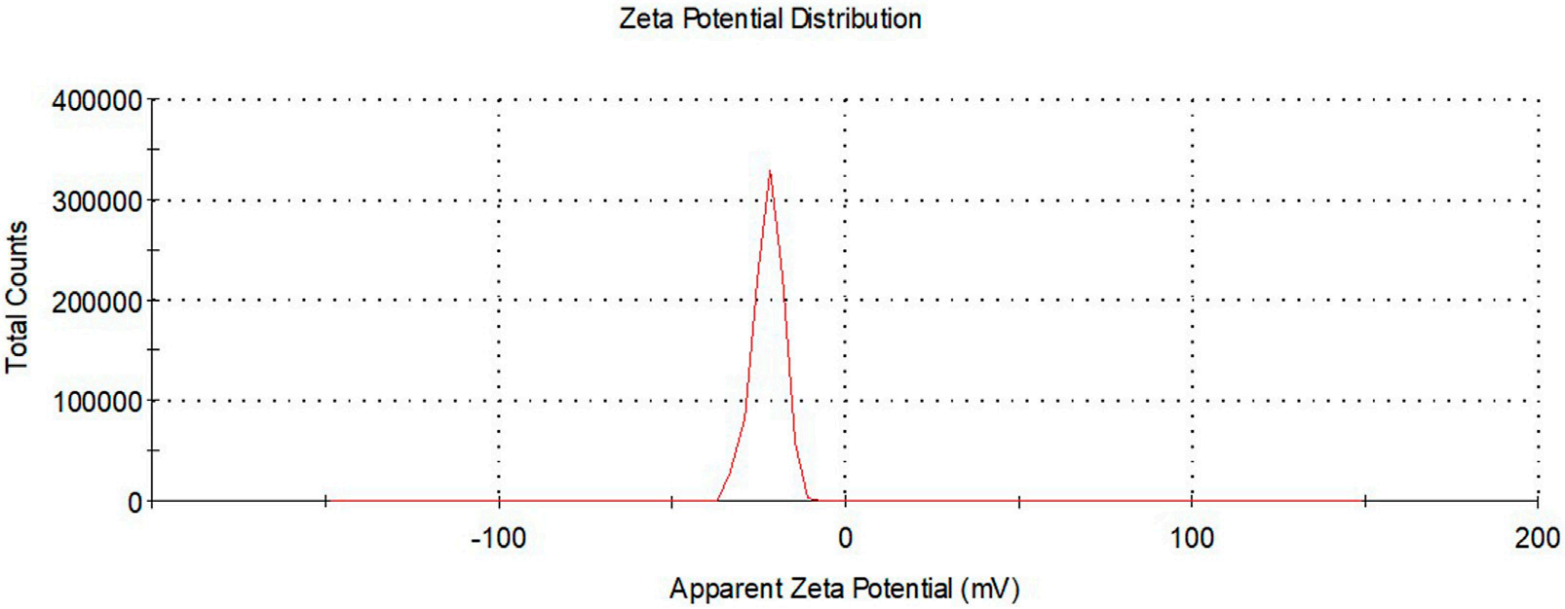
Zeta potential distributions of synthesized AgNPs.

Zeta potential analysis data were evaluated in determining the surface charges of DK-AgNPs synthesized with leaf extract. As seen in Figure 6, the surface charge distribution of the synthesized AgNPs was found to be −22.4 mV on average. The presence of phytochemicals affects the negative surface charge (Remya et al., 2015). The negative charge distribution of NPs is important for their stability. The formation of different charges causes negative situations such as aggregation and fluctuation with electrostatic interaction (Pugazhendhi et al., 2018). The fact that AgNPs synthesized with DK extract were only negatively charged was the data showing that these NPs were stable. When previous Eco-Friendly synthesis studies were examined, it was seen that the surface charge distributions in the zeta potential results of AgNPs were −18.52 mV (Tian et al., 2022), −13.7 mV (Shoaib et al., 2021), and −43.3 mV (Soltani and Darbemamieh, 2021).

### Size distribution of DK-AgNPs

**FIGURE 7:**
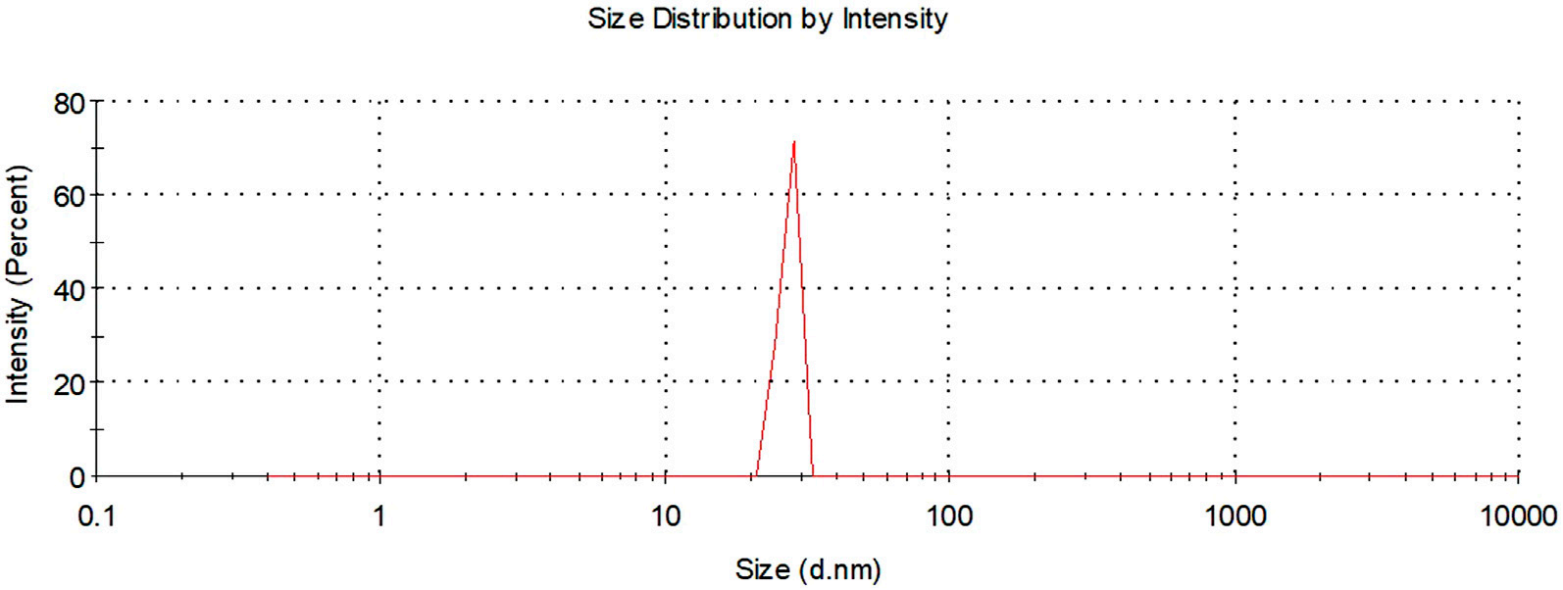
Zeta size distributions of synthesized AgNPs.

The sizes of AgNPs synthesized with DK extract were defined by zeta size distribution analysis. It was observed that AgNPs exhibited an average size distribution of 27.12 nm below 100 nm (Figure 7). It was stated that AgNPs synthesized with *Zataria multiflora* extract at pH 9 had a size distribution of 25 nm (Barabadi et al., 2021). It was shown that AgNPs with an average size distribution of 49.04 nm were synthesized using *Commiphora molmol* extract (Awad et al., 2021).

### TGA-DTA data of DK-AgNPs

**FIGURE 8:**
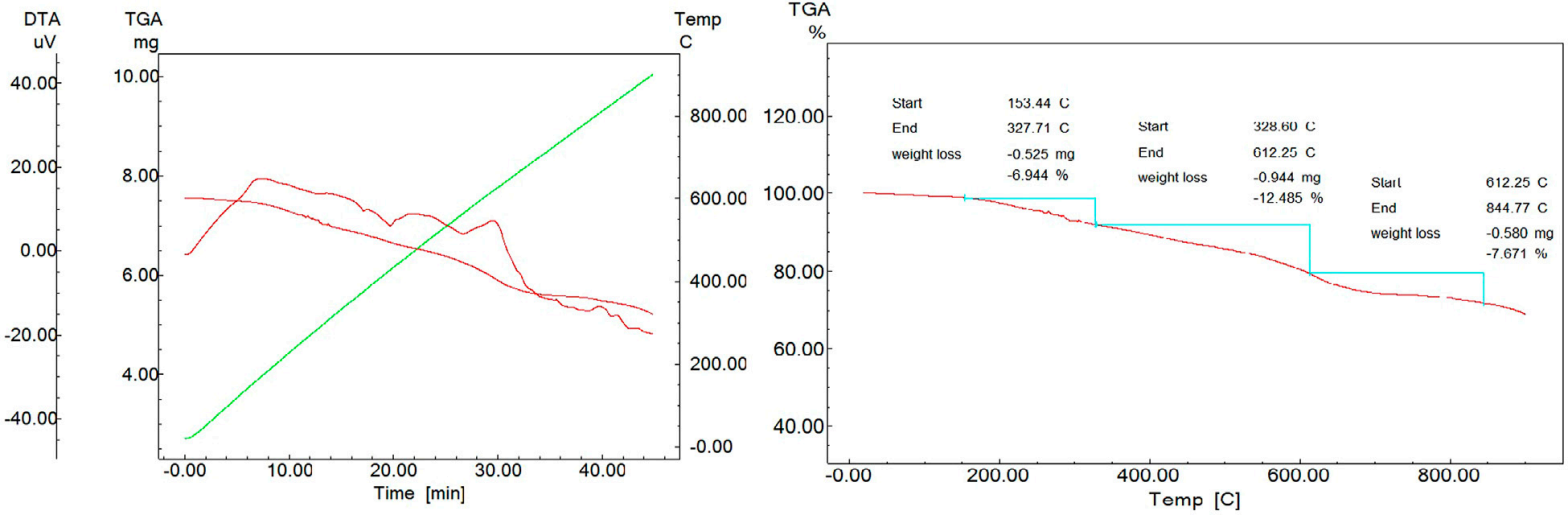
TGA-DTA graph of synthesized AgNPs.

TGA-DTA analysis was performed to determine the stability and resistance of the synthesized AgNPs against heat treatments. As seen in Table 1 and Figure 8, mass losses occurred at temperatures at three points. The initial mass loss from these points was due to the loss of retained water with 6.94% at 153.44°C. It was observed that the mass losses of 12.48% and 7.67% at the second and third points, respectively, were caused by the phytochemicals (organic compounds) surrounding the AgNPs (Mobin et al., 2022). TGA-DT results of AgNPs are given in some green synthesis studies performed in Table 2.

**TABLE 1:** TGA-DTA results and mass loss points of synthesized AgNPs.

Mass loss points	Temperature (°C)	Mass loss (%)
First	153.44–327.71	6.94
Second	328.60–612.25	12.48
Third	612.25–844.77	7.67

**TABLE 2:** Mass loss points in TGA-DTA results of AgNPs synthesized in Eco-Friendly studies.

Biological source	Size (nm)	Mass loss temperature (°C)			Mass loss (%)			References
		1	2	3	1	2	3	
*Cydonia oblonga*	27.30	31–257	328–612	612–844	6.91	12.48	7.67	Mobin et al., 2022
*Fritillaria* (Flower)	10	200	200–600	-	77.5	18.6	-	Hawar et al., 2022
Hawthorn leaves	58.54	11–85	85–235	235–934	2.56	3.56	34.35	Jun et al., 2008
*Crataegus monogyna*								
*Artemisia absinthium*	14.58	30–162	162–478	478–619	1.45	13.18	4.92	Hatipoğlu, (2022)

### FTIR analysis

**FIGURE 9:**
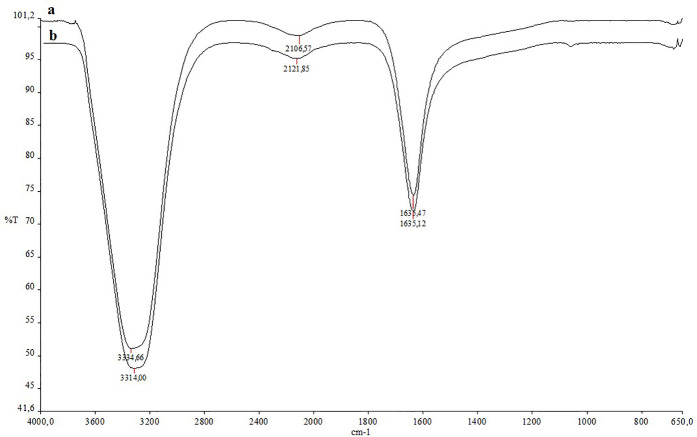
**(A)** FTIR spectroscopy data of the plant extract, **(B)** FTIR spectroscopy data of reaction liquid with synthesized AgNPs.

FTIR analysis was used to characterize the potential functional groups involved in the reduction of silver metal ions to silver nanoparticles. Frequency shifts in 3334.66–3314.00 cm^−1^, 2106.57–2121.85 cm^−1^, and 1635.47–1635.12 cm^−1^ in the results of the FTIR analysis performed to evaluate the functional groups participating in the reaction showed that hydroxyl, carboxyl and amine groups may be responsible for the reduction and stabilization, respectively (Figure 9) (Hatipoğlu, 2022). Proteins and flavonoids may have contributed to the quick reduction and capping of silver ions into silver nanoparticles in the current analysis. Strong reducing agents, flavonoids, which can be indicative of the reduction of silver nitrate to generate AgNPs, were detected in the leaf extract. The decrease of Ag^+^ to Ag^0^ may be directly attributed to the flavonoid components found in the *D. kaki* extract.

### Antimicrobial activities

The antimicrobial effects of AgNPs obtained after synthesis was determined by the microdilution method. The efficacy of synthesised DK-AgNPs (1 μg/mL concentration) was comparing with Ag NO_3_ solution (11.60 μg/mL initial concentration) and antibiotics used as standard (128 μg/mL concentration). As seen in Table 3, concentrations of 0.03 and 0.050 μg mL^−1^ showed suppression of growth of tested strains. These concentrations of AgNPs showed that they were effective at very low concentrations against antibiotics and AgNO_3_ solution. In Table 4, the findings of the antimicrobial effect studies obtained in other Eco-Friendly synthesis studies are given to compare with our results.

**TABLE 3:** MIK values showing inhibition on growth in the antimicrobial effects of synthesized AgNPs.

	DK-AgNPs	Silver nitrate	Antibiotic^a^
	µg ml^-1^	µg ml^-1^	µg ml^-1^
*S. aureus*	0.06	2.65	2.00
*B. subtilis*	0.03	1.32	1.00
*E. coli*	0.25	0.66	2.00
*P. aeruginosa*	0.13	1.32	4.00
*C. albicans*	0.50	0.66	2.00

^a^ The antibiotics used for comparison; are Vancomycin for Gram-positive bacteria, Colistin for negatives, and Fluconazole for *C. albicans*.

**TABLE 4:** Comparative studies of AgNPs on microorganisms in Eco-Friendly synthesis studies.

Biological resource	Size (nm)	Shape	MIC values		References
			µg ml^-1^		
			*S. aureus*	*E. coli*	
Tannic acid and sodium alginate	18.52	Spherical	31.25	-	Tian et al., 2022
*Zataria multiflora*	25.5	Spherical	4–8		Barabadi et al., 2021
Anklet olive tree leaves	7.2	Spherical	0.06	0.13	Ejidike and Clayton, (2022)
Chitosan	<20	Spherical	312.5	39.1	Wongpreecha et al., 2018
*Cynara scolymus* L	28.78	Spherical	0.12	0.13	Baran et al., 2021
*Diospyros kaki* L	27.12	Spherical	0.06	0.25	(This work)

### Cytotoxic effects of DK-AgNPs

The cytotoxic effects of synthesized AgNPs on cell lines were examined using the MTT method. As seen in Table 5, it was determined that 25 μg mL^−1^ concentration did not have a toxic effect on healthy cell lines, and had an anticancer effect on Caco-2 and skov-3 cancer cell lines, with suppression rates of 44.40% and 18.62%, respectively. It was observed that AgNPs at a concentration of 25 μg mL^−1^ showed a proliferative effect on U118. Some concentrations may exert a proliferative effect on cancer cell lines (Zhang and Jiang, 2020). It was determined that 50 μg mL^−1^ concentration of AgNPs did not show any toxic effect in healthy cells and the best anticancer effect of this concentration occurred on the Caco-2 cell line with a suppression rate of 59.49% at 50 μg mL^−1^ concentration (Table 5). In addition, 50 μg mL^−1^ concentration of AgNPs synthesized on U118 and Skov-3 cancer cell lines caused a 16.01% and 51.07% suppression in cell viability and proliferation, respectively.

**TABLE 5:** Viability rates of cell lines after 48 h of the interaction with synthesized AgNPs (*n* = 3).

	Control	25 μg mL^−1^	50 μg mL^−1^	100 μg mL^−1^	200 μg mL^−1^	IC_50_
HDF	100	90.96	51.55	43.83	20.54	1.58
U118	100	108.05	96.37	83.99	81.17	3.73
CaCo-2	100	55.60	40.51	32.74	28.41	3.88
Skov-3	100	81.38	54.59	49.03	44.26	2.97

The cytotoxic effects of AgNPs synthesized in the previous green synthesis studies on cancer cells using the MTT method are given in Table 5. Some properties of nanoparticles such as concentration, shape, and size have a significant effect on their toxic effect (Swamy et al., 2015). NPs contact cells by electrostatic interaction and cause an increase in the amount of ROS DNA, RNA, and some vital enzymes that have a high affinity for these species (Gopu et al., 2022). In addition, AgNPs cause cell death by activating structures responsible for cell apoptosis (Selvan et al., 2018). Some of the studies that evaluated the cytotoxic activities of silver nanoparticles with similar cell lines are given in Table 6 for comparison.

**TABLE 6:** Cytotoxic effects of AgNPs on cancer cell lines in previous green synthesis studies.

	AgNPs				
Cell lines	Shape	Size (nm)	Concentration range (μg mL^−1^)	Viability (%)	References
HDF	Spherical	23.29	25–200	57.47–89.87	Khatamifar et al., 2022
U118	Spherical	7.2	25–200	30.58–59.75	Ejidike and Clayton, (2022)
CaCo-2	Spherical	23.29	25–200	64.99–73.92	Khatamifar et al., 2022
Skov-3	Spherical	162.72	1–40	29.36	Satpathy et al., 2018

### LC-MS profile of DK leaf extract

**FIGURE 10:**
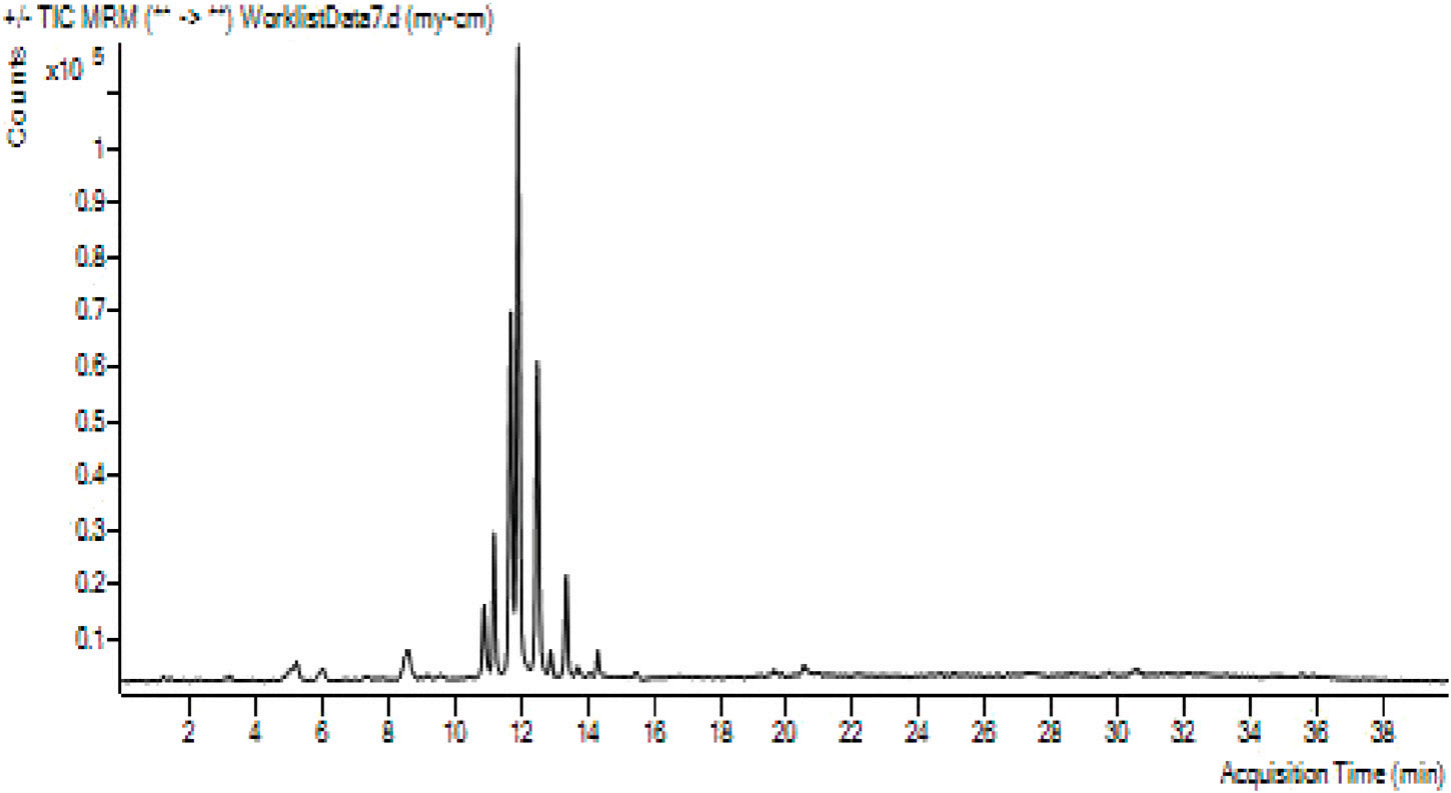
LC-MS/MS profile of the content of DK leaf extract.

Phytochemicals have very important activities such as anticancer, antioxidant, and anti-inflammatory. Phytochemicals such as phenolic compounds and flavonoids are biologically highly active compounds. We take them into our bodies, especially with plant-based foods. LC-MS/MS analysis results for the analysis of phenolic compounds of DK extract were evaluated with the results given in Figure 10 and Table 7. When the compounds with high concentrations in the extract were evaluated, 11210.6471 μg mL^−1^, 13359.8649 μg mL^−1^, 3091.3887 μg mL^−1^, 2100.6615 μg mL^−1^, and 3546.9109 μg mL^−1^ concentrations of compounds such as chlorogenic acid, cynarin, hyperoside, quercetin-3-glucoside, quercetin-3-D-xyloside were determined, respectively. These compounds have very important benefits. It is a member of the ester family of chlorogenic acids between quinic acid and trans-hydroxycinnamic acid. It has pharmacological effects such as antioxidant, antispasmodic, inhibition of DNA methyltransferase, and inhibition of carcinogenic components (Jaiswal et al., 2014). Cynarine (1,3-dicaffeoylquinic acid) is formed by the esterification of two units of caffeic acid and one unit of quinic acid. It is a derivative of hydroxycinnamic acid and is a biologically active functional group. It is a compound with pharmacological effects such as antioxidant, anticholinergic, antihistamine, and antibacterial (Topal et al., 2016). Caffeic acid exhibits antifungal activity via inhibiting fungal 1,3-d-glucan synthase (Mohsin et al., 2022). Quercetin-based compounds in the flavonoid group have antiviral effects. In particular, quercetin-3-glucoside has a strong antiviral effect against the influenza virus (Nile et al., 2020).

**TABLE 7:** LC-MS analysis results for the analysis of phenolic compounds of the plant extract.

Component	RT	Resp	Final concentration (μg mL^−1^)	Unit
Chlorogenic acid	5.23	50527	11210.65 ± 1.79	µg ml^−1^
4-Hydroxybenzaldehyde	5.72	1787	21.37 ± 0.11	µg ml^−1^
Cafeic acid	6.04	26124	251.77 ± 0.23	µg ml^−1^
P-coumaric acid	8.48	5668	34.52 ± 0.09	µg ml^−1^
Transferulic acid	9.56	573	92.48 ± 0.20	µg ml^−1^
Kersimeritrin	10.71	2209	54.18 ± 0.12	µg ml^−1^
Cynarin	11.18	130380	13359.86 ± 1.93	µg ml^−1^
Hyperocyte	11.69	413692	3091.39 ± 0.34	µg ml^−1^
Quercetin-3-glycoside	11.92	833019	2100.66 ± 0.15	µg ml^−1^
Quercetin-3- D-xyloside	12.50	624	3546.91 ± 0.19	µg ml^−1^
Kaemerol-3-glucoside	13.35	109366	178.42 ± 0.08	µg ml^−1^
Fisetin	13.35	1750	153.60 ± 0.09	µg ml^−1^
Biochanin A	20.53	22285	464.49 ± 0.10	µg ml^−1^
Diosgenin	30.53	9053	29.62 ± 0.02	µg ml^−1^

RT, retention time.

## Conclusion

The use of environmentally friendly technology to create nanoparticles has various benefits, including the process’s simplicity and practicality from an economic standpoint. We have created a quick, environmentally friendly, and practical process for creating silver nanoparticles with a diameter range of 8–38 nm from D. kaki aqueous leaf extract. Changes in AgNPs were seen in UV-vis spectra at 453 nm, as well as time-dependent color changes. The XRD pattern indicated that the sample was made up of elemental silver crystalline face-centred cubic (fcc) lattice structures. The essential biological components responsible for the silver reduction in the FTIR technique were discovered. EDX was used to examine the nanoparticles’ high absorption. The SEM image shows the size, shape, and high density of the nanoparticles. All of these findings indicated that AgNPs produced with *Diospyros kaki* leaf extract were stable. The presence of significant phenolic compounds in the LC-MS analysis findings for the detection and identification of phytochemicals of extract components indicated that these components were actively involved in the reaction. In MIC tests, the DK-AgNPs demonstrated significant antibacterial activity. The DK-AgNPs also suppressed the activity of treated cell lines, confirming the viability reduction. The creation of application phases is expected to be beneficial to future research, particularly in the quest for anticancer and antibacterial medicines. Finally, because the synthesis procedure is simple and inexpensive, and the raw material is inexpensive, more *in vivo* tests are required to confirm the results of this work.

## Data Availability

The original contributions presented in the study are included in the article/supplementary material, further inquiries can be directed to the corresponding authors.
